# Supplementary material for: Hot spot prediction in protein-protein interactions by an ensemble system
Source: BMC Syst Biol. 2018 Dec 31;12(Suppl 9):132. doi: 10.1186/s12918-018-0665-8 (PMC6311905; doi:10.1186/s12918-018-0665-8)
Supplement: Supplementary file 2 — All relevant MATLAB code. A simple Matlab implement of our predictor. (ZIP 19,742 kb) [file 12918_2018_665_MOESM2_ESM.zip › Additional file 2/Read ASA/ASA website/SKEMPI/1REWC.htm]

 NetSurfP - Protein Surface Accessibility and Secondary Structure Predictions

  

|  |  |  |
| --- | --- | --- |
|  |  | NetSurfP - Protein Surface Accessibility and Secondary Structure Predictions  Technical University of Denmark |

  


---

```
# For publication of results, please cite:
# A generic method for assignment of reliability scores applied to solvent accessibility predictions.
# Bent Petersen, Thomas Nordahl Petersen, Pernille Andersen, Morten Nielsen and Claus Lundegaard
# BMC Structural Biology 2009, 9:51 doi:10.1186/1472-6807-9-51
#
# Column 1: Class assignment - B for buried or E for Exposed - Threshold: 25% exposure, but not based on RSA
# Column 2: Amino acid
# Column 3: Sequence name
# Column 4: Amino acid number
# Column 5: Relative Surface Accessibility - RSA
# Column 6: Absolute Surface Accessibility
# Column 7: Z-fit score for RSA prediction
# Column 8: Probability for Alpha-Helix
# Column 9: Probability for Beta-strand
# Column 10: Probability for Coil
E T  1REWC                  1    0.860 119.227   0.491   0.003   0.003   0.994
B L  1REWC                  2    0.272  49.876  -0.289   0.018   0.088   0.893
E P  1REWC                  3    0.369  52.347  -0.338   0.019   0.141   0.840
B F  1REWC                  4    0.244  48.971   0.149   0.021   0.451   0.528
B L  1REWC                  5    0.058  10.675  -0.986   0.021   0.756   0.223
E K  1REWC                  6    0.307  63.232   1.024   0.018   0.846   0.136
B C  1REWC                  7    0.023   3.187   0.208   0.011   0.918   0.071
B Y  1REWC                  8    0.286  61.011   0.869   0.002   0.816   0.182
B C  1REWC                  9    0.021   2.976   0.575   0.004   0.514   0.481
E S  1REWC                 10    0.345  40.481   0.361   0.019   0.141   0.840
E G  1REWC                 11    0.726  57.120  -1.638   0.018   0.088   0.893
E H  1REWC                 12    0.437  79.527   0.034   0.018   0.088   0.893
B C  1REWC                 13    0.040   5.644  -0.844   0.018   0.047   0.935
E P  1REWC                 14    0.597  84.700  -0.385   0.018   0.019   0.964
E D  1REWC                 15    0.680  97.974  -1.841   0.018   0.019   0.964
E D  1REWC                 16    0.520  74.990  -1.728   0.018   0.019   0.964
E A  1REWC                 17    0.416  45.788  -1.130   0.018   0.019   0.964
E I  1REWC                 18    0.516  95.460  -0.479   0.053   0.043   0.903
E N  1REWC                 19    0.409  59.892  -1.160   0.018   0.047   0.935
B N  1REWC                 20    0.242  35.458  -1.253   0.052   0.084   0.864
E T  1REWC                 21    0.274  38.018   0.797   0.021   0.451   0.528
B C  1REWC                 22    0.044   6.234  -0.259   0.022   0.552   0.426
E I  1REWC                 23    0.427  79.032   0.893   0.074   0.484   0.442
E T  1REWC                 24    0.476  66.063  -0.756   0.064   0.216   0.721
E N  1REWC                 25    0.624  91.295  -0.677   0.018   0.047   0.935
B G  1REWC                 26    0.256  20.108  -1.190   0.018   0.088   0.893
E H  1REWC                 27    0.309  56.298   0.764   0.022   0.552   0.426
B C  1REWC                 28    0.034   4.802  -0.176   0.021   0.756   0.223
B F  1REWC                 29    0.066  13.306   0.716   0.018   0.846   0.136
B A  1REWC                 30    0.153  16.850   0.637   0.018   0.846   0.136
B I  1REWC                 31    0.127  23.587   0.949   0.011   0.918   0.071
B I  1REWC                 32    0.086  15.910   0.733   0.011   0.918   0.071
B E  1REWC                 33    0.260  45.370   0.294   0.006   0.962   0.032
E E  1REWC                 34    0.369  64.464   0.528   0.011   0.918   0.071
E D  1REWC                 35    0.318  45.766  -0.123   0.023   0.655   0.322
E D  1REWC                 36    0.578  83.319  -0.830   0.021   0.279   0.699
E Q  1REWC                 37    0.716 127.913  -0.815   0.018   0.088   0.893
E G  1REWC                 38    0.483  38.028  -0.464   0.004   0.085   0.910
E E  1REWC                 39    0.408  71.365  -0.035   0.023   0.655   0.322
E T  1REWC                 40    0.371  51.416   0.282   0.011   0.918   0.071
E T  1REWC                 41    0.304  42.109   0.291   0.006   0.962   0.032
B L  1REWC                 42    0.242  44.329   1.081   0.011   0.918   0.071
B A  1REWC                 43    0.311  34.239  -0.748   0.018   0.846   0.136
B S  1REWC                 44    0.202  23.710  -0.003   0.004   0.616   0.381
B G  1REWC                 45    0.239  18.794  -1.720   0.004   0.420   0.576
B C  1REWC                 46    0.055   7.736  -1.332   0.005   0.262   0.733
B M  1REWC                 47    0.126  25.133  -0.257   0.019   0.141   0.840
E K  1REWC                 48    0.287  59.098   0.935   0.005   0.015   0.979
E Y  1REWC                 49    0.363  77.659  -0.154   0.018   0.019   0.964
E E  1REWC                 50    0.663 115.913  -1.180   0.018   0.019   0.964
E G  1REWC                 51    0.633  49.809  -2.363   0.016   0.005   0.979
E S  1REWC                 52    0.435  51.005  -0.994   0.018   0.047   0.935
E D  1REWC                 53    0.380  54.772  -0.070   0.021   0.279   0.699
B F  1REWC                 54    0.070  14.009  -0.413   0.021   0.451   0.528
B Q  1REWC                 55    0.271  48.365   0.245   0.023   0.655   0.322
B C  1REWC                 56    0.030   4.226  -0.978   0.023   0.655   0.322
E K  1REWC                 57    0.378  77.713   0.369   0.022   0.359   0.619
E D  1REWC                 58    0.519  74.817  -1.593   0.019   0.141   0.840
E S  1REWC                 59    0.400  46.903   0.094   0.019   0.141   0.840
E P  1REWC                 60    0.443  62.805  -1.293   0.018   0.088   0.893
E K  1REWC                 61    0.454  93.408  -0.694   0.018   0.047   0.935
E A  1REWC                 62    0.421  46.372  -0.876   0.018   0.047   0.935
E Q  1REWC                 63    0.438  78.155  -0.176   0.018   0.047   0.935
E L  1REWC                 64    0.450  82.340  -1.749   0.018   0.047   0.935
E R  1REWC                 65    0.484 110.928  -0.054   0.019   0.141   0.840
E R  1REWC                 66    0.318  72.868  -0.041   0.022   0.552   0.426
B T  1REWC                 67    0.198  27.393   1.341   0.018   0.846   0.136
B I  1REWC                 68    0.169  31.247   0.413   0.018   0.846   0.136
B E  1REWC                 69    0.125  21.785   0.254   0.018   0.846   0.136
B C  1REWC                 70    0.025   3.482   0.345   0.023   0.655   0.322
B C  1REWC                 71    0.251  35.198   0.713   0.019   0.141   0.840
E R  1REWC                 72    0.638 146.125  -0.308   0.018   0.019   0.964
E T  1REWC                 73    0.505  70.030  -0.356   0.018   0.019   0.964
E N  1REWC                 74    0.514  75.294  -1.093   0.018   0.019   0.964
B L  1REWC                 75    0.181  33.178   0.727   0.053   0.043   0.903
B C  1REWC                 76    0.053   7.399  -0.375   0.053   0.043   0.903
B N  1REWC                 77    0.246  35.941  -0.433   0.053   0.043   0.903
E Q  1REWC                 78    0.550  98.230  -0.303   0.053   0.043   0.903
E Y  1REWC                 79    0.419  89.498  -0.297   0.052   0.084   0.864
B L  1REWC                 80    0.094  17.138   0.352   0.018   0.088   0.893
E Q  1REWC                 81    0.432  77.173   0.500   0.004   0.085   0.910
B P  1REWC                 82    0.227  32.197  -0.821   0.005   0.045   0.951
E T  1REWC                 83    0.589  81.708  -0.959   0.004   0.085   0.910
B L  1REWC                 84    0.223  40.776   0.105   0.005   0.045   0.951
E P  1REWC                 85    0.446  63.273   0.301   0.005   0.015   0.979
E P  1REWC                 86    0.787 111.619  -1.805   0.000   0.000   0.999

Explain the output.  Go back.
```

---
